# Case report: First isolation of *Exophiala dermatitidis* from subcutaneous phaeohyphomycosis in a cat

**DOI:** 10.3389/fvets.2023.1259115

**Published:** 2023-09-18

**Authors:** Hironari Osada, Maiko Nagashima-Fukui, Taiga Okazawa, Miki Omura, Koichi Makimura, Keitaro Ohmori

**Affiliations:** ^1^Animal Medical Center, Tokyo University of Agriculture and Technology, Fuchu, Tokyo, Japan; ^2^Cooperative Department of Veterinary Medicine, Faculty of Agriculture, Tokyo University of Agriculture and Technology, Fuchu, Tokyo, Japan; ^3^School of Medicine, Graduate School of Medicine, Teikyo University, Itabashi, Tokyo, Japan; ^4^Mycolabo Co., Ltd, Mitaka, Tokyo, Japan; ^5^Teikyo University Institute of Medical Mycology, Hachioji, Tokyo, Japan; ^6^Division of Animal Life Science, Institute of Agriculture, Tokyo University of Agriculture and Technology, Fuchu, Tokyo, Japan

**Keywords:** antifungal susceptibility test, cat, *Exophiala dermatitidis*, fungus, phaeohyphomycosis

## Abstract

Phaeohyphomycosis, which is caused by the opportunistic black yeast-like fungus *Exophiala dermatitidis*, has been reported in humans and dogs. However, no previous studies describing *E. dermatitidis* infections in cats have been published. Herein, we report a case of subcutaneous phaeohyphomycosis caused by *E. dermatitidis*. A 12-year-old, castrated male Japanese domestic short-haired cat presented with a solitary subcutaneous abscess on the left side of the neck, where an esophageal tube for force-feeding had been placed previously. The cat was diagnosed with hepatitis and was treated with prednisolone. The subcutaneous abscess was incised using a scalpel blade and the pus was excreted. The cytology of the pus revealed hyphae with neutrophil and macrophage infiltration. Although the cat was treated with oral itraconazole or an infusion of topical ketoconazole cream applied to the lesion, it died. The fungal culture of the pus specimen developed dark-green, waxy, smooth, yeast-like colonies. Sequencing of the internal transcribed spacer 1–4 regions of the ribosomal DNA of the pus specimen showed 100% identity with that of the standard strains of *E*. *dermatitidis*. Based on these results, the cat was diagnosed with subcutaneous phaeohyphomycosis caused by *E*. *dermatitidis*. The antifungal susceptibility test revealed that the fungus showed low or moderate susceptibility to the antifungal drugs examined, except for amphotericin B, which exhibited high *in vitro* antifungal activity. This is the first case report to provide definitive evidence of *E*. *dermatitidis* infection in cats and antifungal susceptibility test results against clinically isolated *E*. *dermatitidis*.

## 1. Introduction

*Exophiala dermatitidis* is an opportunistic black yeast-like fungus responsible for phaeohyphomycosis (1). *E. dermatitidis* is dark pigmented by melanin, a component of the cell wall (1). It is a globally distributed and ubiquitous fungus, but is rarely isolated from the environment (2). It is frequently found in hot and moist man-made indoor habitats such as dishwashers, steam baths, and sauna facilities (1). Although *E. dermatitidis* infections are rare, *E. dermatitidis* causes phaeohyphomycosis in immunosuppressed and immunocompetent individuals (1). The most common manifestations of phaeohyphomycosis are cutaneous and subcutaneous infections, but the fungus less frequently leads to systemic dissemination involving extracutaneous organs such as the central nervous system, heart, gastrointestinal tract, lungs, and bones (3). Once *E. dermatitidis* infections are established, aggressive combined therapies involving surgical excision and antifungal drugs are required (3).

In small animal practice, *E. dermatitidis* infections have been reported in two dogs (4, 5). *E. dermatitidis* formed multiple black or purple subcutaneous nodules on the neck of a dog with a history of multicentric lymphoma that had received chemotherapy for four months (4). In the other dog diagnosed with atopy and treated with oral ciclosporin for six months, *E. dermatitidis* induced an intra-abdominal mass with lymphadenopathy (5). Although *E. dermatitidis* infection in a cat has been reported as a congress abstract (6), no peer-reviewed studies describing *E. dermatitidis* infections in cats have been published. Here, we report subcutaneous phaeohyphomycosis caused by *E. dermatitidis* infection in a cat with hepatitis during prednisolone treatment.

## 2. Case description

**Figure 1:**
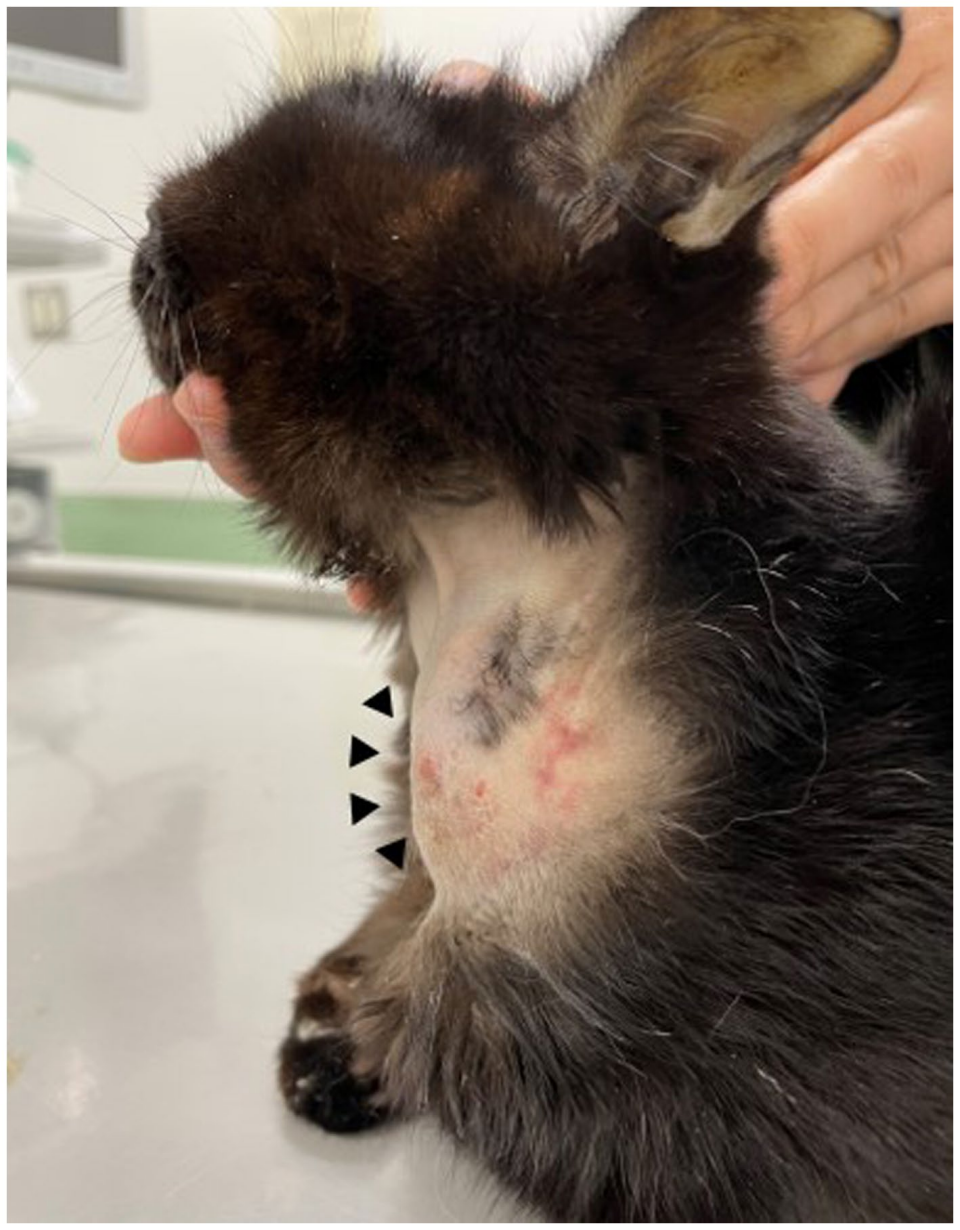
Gross appearance of a subcutaneous abscess (arrowheads) on the left neck.

A 12-year-old, castrated male Japanese domestic short-haired cat weighing 2.0 kg visited the Tokyo University of Agriculture and Technology Animal Medical Center because of a solitary subcutaneous soft mass on the left side of the neck on day 1 (Figure 1). The cat was kept indoors with other two cats. The cat’s owner noticed a mass approximately 1 cm in diameter three days prior, and the size of the mass increased until the visit. Because the cat had a history of chronic kidney disease (CKD), severe anorexia, elevated liver enzymes, and jaundice, an esophageal tube for force-feeding was placed 76 days prior on the left side of the neck where the mass was found. In addition, the cat was diagnosed with suppurative lymphoplasmacytic cholangiohepatitis by laparoscopic liver biopsy 49 days before presentation. The esophageal tube was removed during laparoscopic liver biopsy due to improvement in anorexia. The bacterial culture of bile collected during laparoscopic liver biopsy was negative. Thus, the cat was administered prednisolone (prednisolone; Teva Takeda Yakuhin Ltd., Aichi, Japan, 1–2 mg/kg, *per os* [PO], q24h) until day 1. Anorexia, elevated liver enzymes, and jaundice were well controlled with prednisolone treatment.

**Figure 2:**
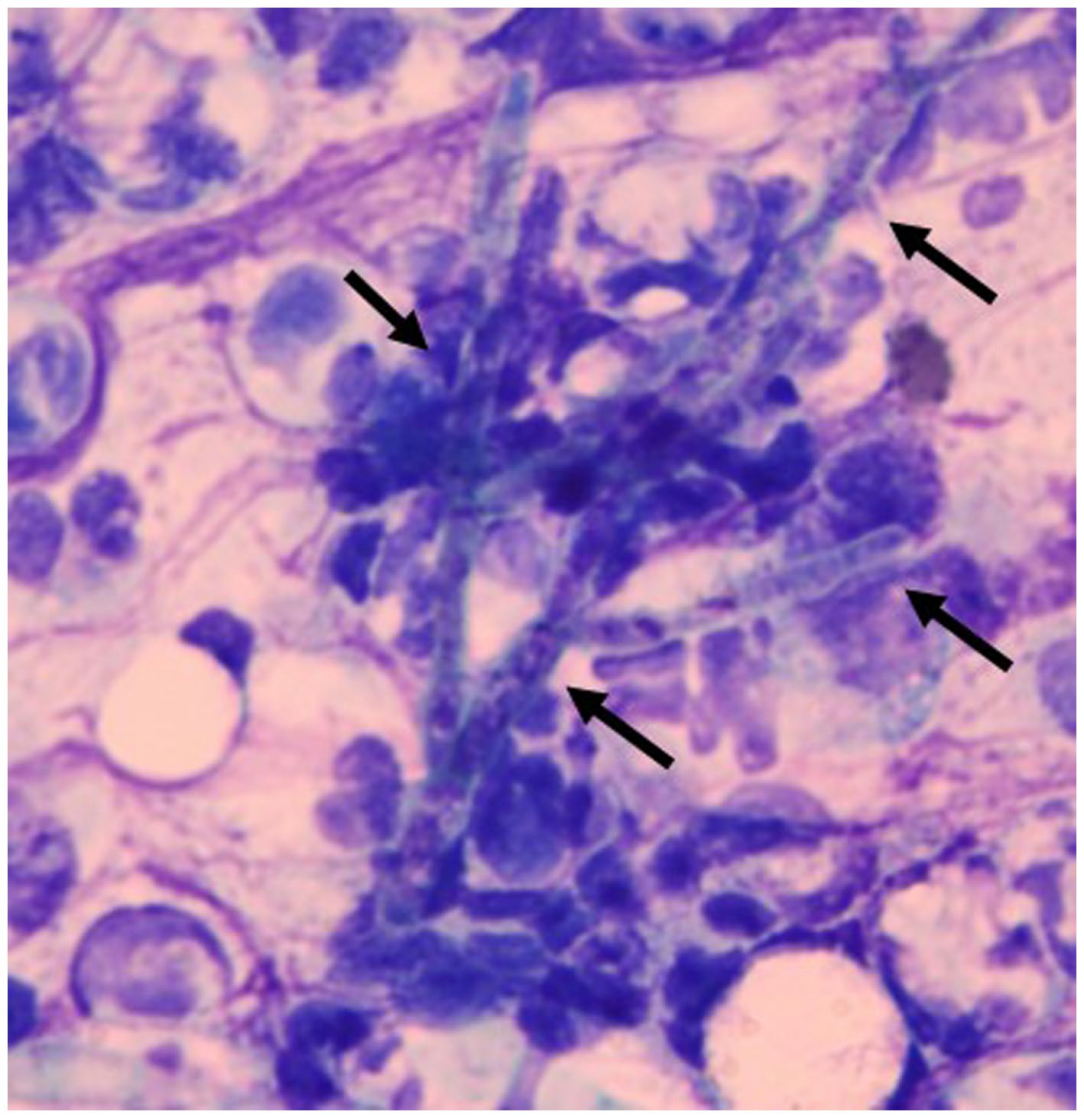
Smear of the pus excreted from a subcutaneous abscess. Hyphae (allows) were observed with neutrophil and macrophage infiltration. Diff-Quik staining. ×1,000.

On day 1, the subcutaneous mass was incised using a scalpel blade and the pus was excreted. Cytological examination of the pus revealed hyphae with neutrophil and macrophage infiltration (Figure 2). Thus, the mass was diagnosed as an abscess. The bacterial culture of the pus was negative. On day 3, local treatment with an incision of the subcutaneous abscess and washing with saline and systemic administration of itraconazole (Itrizole capsules; Janssen Pharmaceutical K.K., Tokyo, Japan, 10 mg/kg, PO, q24h) were initiated. However, the owner observed anorexia and lethargy soon after treatment initiation. Although the plasma total bilirubin (TBIL) level was 0.7 mg/dL (reference interval [RI]: 0.1–0.5 mg/dL) 23 days prior to treatment, it was elevated three days after initiation (1.8 mg/dL). Because these abnormalities were considered to be induced by the adverse effects of itraconazole, the drug was discontinued on day 5. Subsequently, local treatment with incision, washing, and infusion of topical ketoconazole cream (Nizoral Cream 2%; Janssen Pharmaceutical K.K.) into the subcutaneous abscess was conducted every 2–3 days. Prednisolone (1 mg/kg, PO, q24h) was also continued as treatment for the underling hepatitis. The cat was locally managed for several weeks. However, the lesion enlarged again, and the general condition gradually worsened. On day 30, aggravation of anorexia and lethargy, azotemia (blood urea nitrogen, 80.7 mg/dL; RI, 17.6–32.8 mg/dL), hypokalemia (K, 2.4 mEq/L; RI, 3.4–4.6 mEq/L), and jaundice (TBIL, 5.2 mg/dL) manifested. Despite intensive care management, the cat died on day 31.

**Figure 3:**
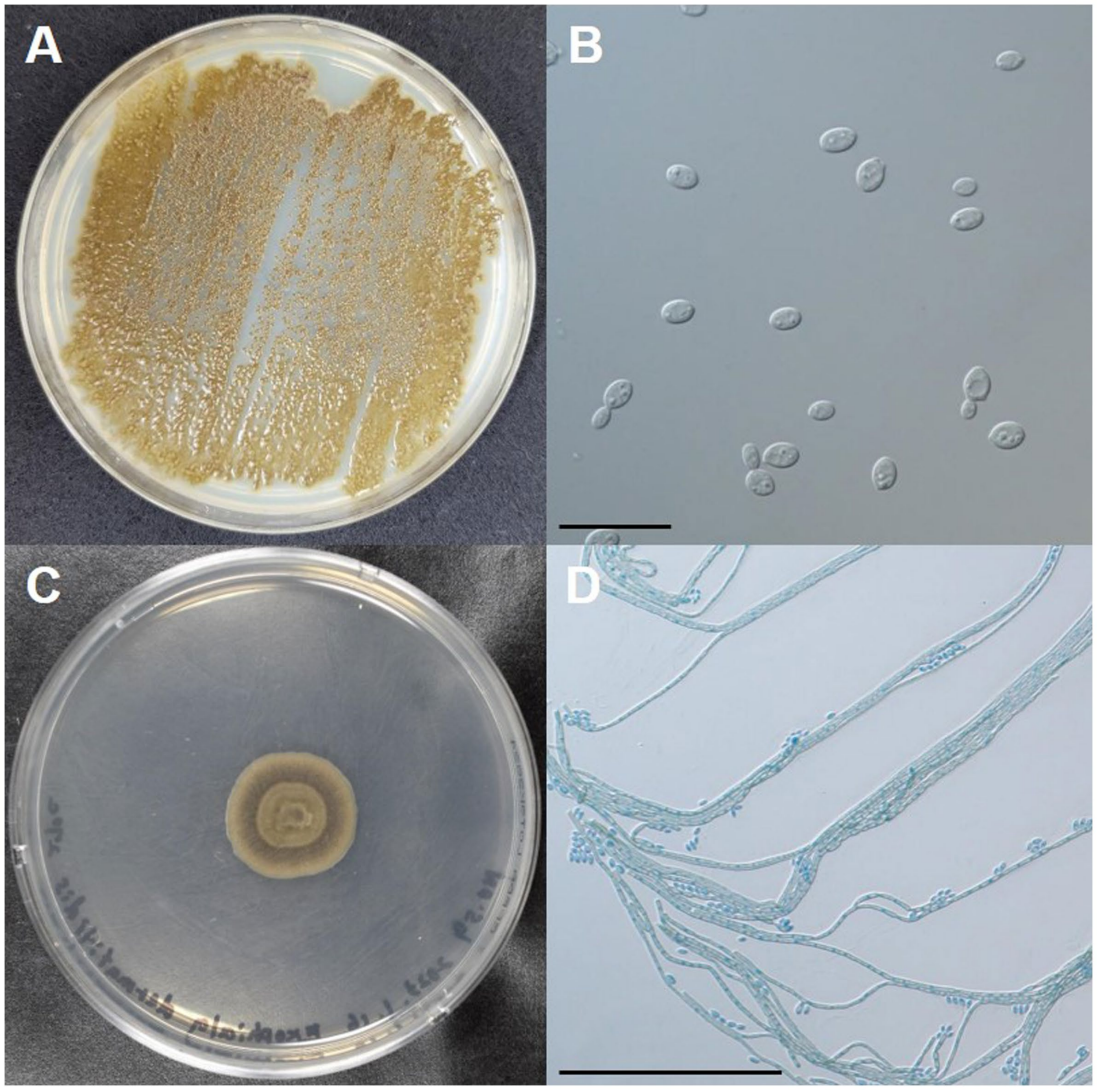
Macro- and micro-morphologies of the clinical isolate of *Exophiala dermatitidis* from a cat with subcutaneous phaeohyphomycosis. **(A)** Colonies incubated on potato dextrose agar (PDA) at 35°C for 7 days. **(B)** Budding oval yeasts were observed under a microscope. Scale bars: 20 μm. **(C)** A colony incubated on PDA at 27°C for 7 days. **(D)** Brown septate hyphae produced conidiospores laterally or on the apex. Scale bars: 100 μm.

To identify the fungus, the pus collected from the subcutaneous abscess was inoculated onto a potato-dextrose agar plate (PDA) and incubated for 7 days at 35°C, 27°C, and 42°C. Morphologically, dark-green, waxy, smooth, yeast-like colonies developed at 35°C (Figure 3A). Budding oval yeasts were observed under a microscope (Figure 3B). A dark-green colony with aerial mycelium developed at 27°C (Figure 3C). Microscopic examination revealed brown septate mycelia and oval conidiophores (Figure 3D). Colonies showed yeast-like development even at 42°C. For molecular identification of the fungus, DNA was extracted from the isolate using the QIAamp DNA Mini Kit (QIAGEN, Hilden, Germany) and sequenced for the internal transcribed spacer 1–4 regions of ribosomal DNA as described previously (7, 8). DNA sequencing results were 100% identical to the standard strains of *E*. *dermatitidis* (Fungal Biodiversity Centre [CBS] 125841, GenBank accession number, MH863897; CBS 120454, MH863085). Based on these results, the cat was diagnosed with subcutaneous phaeohyphomycosis caused by *E*. *dermatitidis*.

The antifungal susceptibility of 10 drugs on the isolated *E*. *dermatitidis* was assessed using a modified broth microdilution method based on the Clinical and Laboratory Standards Institute M38 3rd edition (Table 1) (9). To date, no validated breakpoints have been established for antifungal drugs against *E*. *dermatitidis*. Of the 10 antifungal drugs, the endpoints for the minimum inhibitory concentrations (MICs) of amphotericin B, flucytosine, fluconazole, itraconazole, terbinafine, and voriconazole and the minimum effective concentrations (MECs) of micafangin and caspofungin were defined in the document (9). In contrast, the endpoints for the MICs of ketoconazole and miconazole were not established in the document. Therefore, we defined the MICs of these drugs as the lowest concentrations that resulted in 80% growth inhibition. The MICs and MECs of the 10 drugs are shown in Table 1. The antifungal susceptibility test results revealed that amphotericin B (MIC, 0.12 μg/mL) displayed high *in vitro* activity against *E*. *dermatitidis*. Considering the tested concentrations of each antifungal drug, itraconazole (MIC, 0.25 μg/mL) and ketoconazole (MIC, 0.5 μg/mL) had moderate *in vitro* antifungal activity against the fungus. However, the fungus showed low susceptibility to other antifungal drugs examined (Table 1).

**Table 1:** The antifungal susceptibility test on the clinical isolate of *Exophiala dermatitidis* from a cat with subcutaneous phaeohyphomycosis.

Antifungal drugs	MIC or MEC (μg/mL)	Tested concentrations (μg/mL)
Amphotericin B	0.12	0.03–16
Fluconazole	8	0.12–64
Flucytosine	4	0.12–64
Itraconazole	0.25	0.015–8
Ketoconazole	0.5	0.03–16
Miconazole	1	0.03–16
Terbinafine	2	0.004–2
Voriconazole	1	0.015–8
Caspofungin	16	0.03–16
Micafangin	4	0.03–16

Minimum inhibitory concentration (MIC): amphotericin B, fluconazole, flucytosine, itraconazole, ketoconazole, miconazole, terbinafine, and voriconazole; minimum effective concentration (MEC): caspofungin and micafangin.

## 3. Discussion

Although feline *Exophiala* spp. infections, including *E. attenuata*, *E. jeanselmei*, and *E. spinifera*, have been reported previously (10–18), this is the first definitive report of the isolation and molecular identification of *E*. *dermatitidis* in a cat with subcutaneous phaeohyphomycosis. This report also revealed *in vitro* activities of antifungal drugs against clinically isolated *E*. *dermatitidis*. *E. dermatitidis* is an emerging opportunistic fungus found in humans and dogs (1–5). Although little is known about the transmission routes of *E*. *dermatitidis* even in humans and dogs, it should be noted that *E. dermatitidis* can infect cats and aggressive treatment is required for infected cats.

*E. dermatitidis* infections have been reported in both immunocompromised and immunocompetent humans, but underlying diseases and immunosuppressive conditions, such as glucocorticoid treatment, cancer, or transplantation, pose risks for *E. dermatitidis* infections in humans (3). The cat described in this report was treated with prednisolone for hepatitis. Furthermore, *E. dermatitidis* infection developed at the site of esophageal feeding-tube removal in the cat. Considering the fungal characteristics, it is highly possible that hepatitis as an underlying disease, esophageal feeding-tube placement, and immunocompromised conditions induced by long-term prednisolone treatment triggered *E. dermatitidis* infection in this cat. Indeed, the other two immunocompetent cats living with this cat did not show any clinical signs associated with *E. dermatitidis* infection.

Previous studies investigated the *in vitro* activities of various antifungal drugs against clinical and environmental isolates of *E*. *dermatitidis* in humans (1). However, the MICs and MECs of antifungal drugs varied, and no significant differences have been detected in the antifungal activities (1). Thus, optimal antifungal drugs against *E*. *dermatitidis* have not yet been identified. In veterinary medicine, no studies have analyzed the *in vitro* activities of antifungal drugs against *E*. *dermatitidis*. In this report, we revealed that the clinical isolate of *E*. *dermatitidis* from the cat was susceptible to amphotericin B. Itraconazole and ketoconazole showed moderate *in vitro* activities against this fungus. These findings are consistent with those previously reported in humans (1). The remaining antifungal drugs (fluconazole, flucytosine, miconazole, terbinafine, voriconazole, caspofungin, and micafungin) showed high MICs and MECs, suggesting that the clinical isolate was resistant to these drugs. Among these drugs, the high MIC of terbinafine in this report (2 μg/mL) was contrary to the previous findings that showed a low MIC of terbinafine against clinical isolates of *E*. *dermatitidis* in humans (≤ 0.01 μg/mL) (1). Given the varied MICs and MECs of antifungal drugs against *E*. *dermatitidis* in humans and among species, the results of antifungal activities in this report may not be applicable to other cats with *E*. *dermatitidis* infections. Antifungal drugs should be selected based on their *in vitro* activities against *E*. *dermatitidis* in individual cases.

Successful treatment outcome has been reported in a dog with intra-abdominal *E. dermatitidis* infection by a combination of surgery and oral administration of voriconazole and terbinafine (5). The cat in this report was administered itraconazole for three days; however, we could not continue to use this drug because of a presumptive adverse event in the liver. Amphotericin B was an alternative based on its *in vitro* antifungal activity against the clinical isolate of *E. dermatitidis*. Since amphotericin B induces renal toxicity as an adverse event, it was impractical to choose this drug because of the history of CKD and the occurrence of azotemia in the cat. In addition, voriconazole and terbinafine showed low *in vitro* antifungal activities against the clinical isolate of *E. dermatitidis* from the cat (MIC, 1 and 2 μg/mL, respectively). In this report, we performed a combination of local therapies, including pus excretion through a small incision, saline washing, and ketoconazole cream infusion, to the subcutaneous lesion. However, local treatment did not achieve remission, and the cat died. As treatment for local lesions associated with feline phaeohyphomycosis not limited to *E. dermatitidis*, surgical excision and systemic administration of antifungal drugs, including itraconazole, posaconazole, and amphotericin B, have been suggested (19). In this report, we did not investigate the antifungal effect of posaconazole, but it was shown to be active against clinical isolates of *E. dermatitidis* from humans (MIC, < 0.002–0.25 μg/mL) (1). Thus, it is worth testing the antifungal activity of posaconazole against feline isolates of *E. dermatitidis*. Because treatment of *E. dermatitidis* infections is often challenging, even in humans, further studies are warranted in veterinary medicine.

The cause of death in this case was unknown; however, disseminated *E. dermatitidis* infection may have been implicated based on the clinical course. Although disseminated *E. dermatitidis* infection is rare in humans, it is more common in immunocompromised patients (3). The cat in this report was immunocompromised due to long-term prednisolone treatment. Thus, after local subcutaneous *E. dermatitidis* infection, disseminated infection may have occurred and invaded the visceral organs, including the liver, in the cat. Alternatively, the cat may have already been infected with *E. dermatitidis* systemically through the esophageal tube, and prednisolone treatment may have triggered local and systemic clinical signs. In such cases, aggressive examinations, including blood cultures for *E. dermatitidis*, would be useful for determining disseminated infection.

In conclusion, the present case report provides evidence of *E. dermatitidis* infection in the cat. Because *E. dermatitidis* is a ubiquitous fungus, the prevalence of *E. dermatitidis* infections in cats should be investigated. In addition, to develop therapeutic strategies for feline *E*. *dermatitidis* infections, the etiology and pathogenicity of this fungus in cats must be elucidated.

## Data availability statement

The original contributions presented in the study are included in the article/supplementary material, further inquiries can be directed to the corresponding author.

## Ethics statement

Ethical approval was not required for the studies involving animals in accordance with the local legislation and institutional requirements because this is a case report of a cat. Written informed consent was obtained from the owner for the participation of the animal in this study.

## Author contributions

HO: Data curation, Formal analysis, Investigation, Writing – original draft. MN-F: Data curation, Investigation, Writing – review & editing. TO: Data curation, Investigation, Writing – review & editing. MO: Formal analysis, Investigation, Writing – original draft, Writing – review & editing. KM: Writing – review & editing. KO: Conceptualization, Data curation, Formal analysis, Investigation, Methodology, Project administration, Supervision, Validation, Writing – original draft, Writing – review & editing.

## Funding

The author(s) declare that no financial support was received for the research, authorship, and/or publication of this article.

## Conflict of interest

MO was employed by Mycolabo Co., Ltd.

The remaining authors declare that the research was conducted in the absence of any commercial or financial relationships that could be construed as a potential conflict of interest.

## Publisher’s note

All claims expressed in this article are solely those of the authors and do not necessarily represent those of their affiliated organizations, or those of the publisher, the editors and the reviewers. Any product that may be evaluated in this article, or claim that may be made by its manufacturer, is not guaranteed or endorsed by the publisher.
